# Dietary Sodium Intake and Food Sources among Chinese Adults: Data from the CNNHS 2010–2012

**DOI:** 10.3390/nu12020453

**Published:** 2020-02-11

**Authors:** Kehong Fang, Yuna He, Yuehui Fang, Yiyao Lian

**Affiliations:** National Institute for Nutrition and Health, Chinese Center for Disease Control and Prevention, No. 29 Nanwei Road, Xicheng District, Beijing 100050, China; kehongsky@163.com (K.F.); fangyh@ninh.chinacdc.cn (Y.F.); lian_yiyao@163.com (Y.L.)

**Keywords:** sodium, salt, dietary, Chinese

## Abstract

The present study was done to examine the status of dietary sodium intake and dietary sources of sodium among Chinese adults. Data were obtained from China National Nutrition and Health Surveillance (CNNHS) 2010–2012. All adults recruited in this study provided complete dietary data on three-day consecutive 24-h dietary recalls combining with the household weighing method. Sodium intake was adjusted for energy to 2000 kcal/day using the residual method. Average sodium intake was 5013 (95% Confidence Interval, CI: 4858, 5168) mg/day, and 92.6% of adults’ sodium intake exceeded the standard in the Chinese proposed intake for preventing non-communicable chronic diseases (PI-NCD). The salt added to food was the main contributor to daily sodium intake, representing 69.2% of the total sodium consumption. The proportion of sodium from salt was different in some subgroups. The contribution ranged from 64.8% for those who came from urban areas aged 18–49 years old to 74.7% for those who came from rural areas with education levels of primary school or less, and sodium from soy sauce was the next highest contributor (8.2%). The proportion of the subjects with sodium intake contributed by flour products was higher in the north with 7.1% than the south with 1.4%. The average consumption of sodium among Chinese was more than the recommended amount, and salt was the main source of sodium.

## 1. Introduction

The sodium intake recommended by The World Health Organization (WHO) is below 2000 mg/day to prevent chronic disease [1]; however, only a few countries have a daily dietary sodium consumption that does not exceed the recommended level around the world [2]. Overconsumption of dietary sodium is the top risk factor for cardiovascular diseases, such as hypertension, stroke and heart failure [3,4,5,6,7] which are the major causes of death and disability in China and the rest of the world [8,9]. In China, high sodium intake is the leading dietary risk factor for cardiometabolic mortality and was associated with a population attributable fraction (PAF) of 17.3% in 2010 [10]. The global burden of disease (GBD) study also showed that 3 million people died from overconsumption of sodium in 2017 around the world [11]. Overconsumption of sodium is one of the most important factors affecting the global burden of disease. Thus, some countries regard sodium intake as a major public health concern.

Some countries have developed strategies to limit sodium intake to meet the recommended consumption set by WHO [12,13,14,15]. Because the lack of knowledge on dietary sodium intake as well as food sources are major obstacles in controlling sodium consumption, some surveys were conducted to estimate the sodium intake and source of sodium in adults with different dietary habits. For example, the consumption of sodium in the United Kingdom and Japan is 3406 mg/day and 4651 mg/day, respectively. The majority of sodium comes from processed foods (95%) in the United Kingdom, and the contribution of soy sauce and salted food to daily sodium intake is about 63% in Japan [16]. However, there is scarce information about the status of dietary sodium intake and dietary sources of sodium in China, and sodium consumption by the Chinese is also different from that in other countries due to the different dietary structures [13,17]. Therefore, understanding sodium consumption and the sources of dietary sodium among Chinese people would be helpful in developing preventive strategies to reduce sodium intake.

## 2. Materials and Methods

### 2.1. Study Population

This cross-sectional analysis was based on data from the China National Nutrition and Health Surveillance (CNNHS). The CNNHS was carried out on a stratified, multistage, systematic, clustered, and random sampling method, proportional to the population, to form a representative sample of China as a whole, including 31 provinces (excluding Hong Kong, Macao, and Taiwan). Firstly, according to the level of economic development, administrative units at the county level in China are divided into four categories: large cities, small and medium-sized cities, ordinary rural areas, and poor rural areas. In the end, 150 monitoring sites were selected in China, including 34 large cities, 41 small and medium-sized cities, 45 ordinary rural areas and 30 poor rural areas; secondly, according to the composition ratio of urban and rural populations in the selected monitoring sites, 6 residential (village) committees were selected from each monitoring site, and 75 households were selected from the selected residential (village) committees by a simple random sampling method, 30 of which were dietary survey households. The survey was approved by the Ethical Committee of the National Institute for Nutrition and Food Safety at the Chinese Center for Disease Control and Prevention (2013(018)) [18].

### 2.2. Estimating Total Sodium Intake

The questionnaire was designed to collect information about socio-demographics, diet, health, and lifestyle status. Well-trained dietary staff collected individual dietary sodium intake over a consecutive three-day, 24-h dietary recall combined with a household food weighing method (including two weekdays and one weekend day). The amount of edible oil and ingredients (such as salt, soy sauce, chicken essence, and other condiments) used at home were measured by a uniformly calibrated electronic scale. Dietary sodium intake from each food was calculated according to the Chinese Food Composition Table [19], and the food not included in the table was grouped into the most similar categories. The Chinese proposed intakes for preventing non-communicable chronic diseases (PI-NCD) cut-point was used to estimate the population prevalence of excess intake. The overconsumption of PI-NCD sodium was defined as dietary sodium ≥2000 mg/day for 18 to 49 years old, ≥1900 mg/day for 50 to 64 years old, or ≥1800 mg/day for 65 years old and over [20]. The sodium intake recommended by The World Health Organization (WHO) is below 2000 mg/day [1]. Sodium intake was adjusted for energy to 2000 kcal/day using the residual method [21].

### 2.3. Statistical Analysis

All analyses were carried out with SAS 9.4 (SAS Institute, Cary, NC, USA). Applying the post-stratification population sampling weights derived for the dietary surveys from the sampling probability of the 2010 Chinese population aged 18 years or older (based on census data), we estimated the nationally representative population levels for intake of food and nutrients. Data are expressed as mean (95% CI) and weighted percent, and differences between groups were analysed using survey means or weighted percentages for survey design. A *p* value < 0.05 was considered to be statistically significant.

## 3. Results

### 3.1. Study Population Characteristics

The CNNHS recruited 53,993 adults with complete dietary intake data (Table 1), 25,309 (46.9%) of whom were 18–49 years old, 18,320 (33.9%) were 50–64 years old, and 10,364 (19.2%) were 65 years old and over. Males accounted for 24,777 (45.9%), and 25,212 (46.7%) of subjects were from the north, 26,782 (49.6%) of whom were urban subjects and 27,211 (50.4%) were rural subjects.

### 3.2. Dietary Sodium Intakes of the Subjects

The average sodium consumption was 5013 (95% CI: 4858, 5168) mg/day for the study adults, and there was a higher consumption among urban (5060 mg/day) compared with rural (4966 mg/day) adults. Urban adults in the south consumed more sodium (mean = 4925 mg/day, 95% CI: 4736, 5114) than rural adults (mean = 4408 mg/day, 95% CI: 4100, 4717). On the contrary, the urban adults in the north (mean = 5238 mg/day, 95% CI: 4931, 5544) consumed less sodium than rural adults (mean = 5544 mg/day, 95% CI: 5210, 5877). The medians of sodium intake were higher than the specific PI-NCD for each respective age group, and the records were 4439 mg/day in 18–49 years old, 4702 mg/day in 50–64 years old, and 4488 mg/day in over 65 years old. The prevalence of sodium intake above PI-NCD among all adults included in the study was 92.6% (Table 2).

### 3.3. Proportion of Dietary Sodium from Various Sources

**Figure 1:**
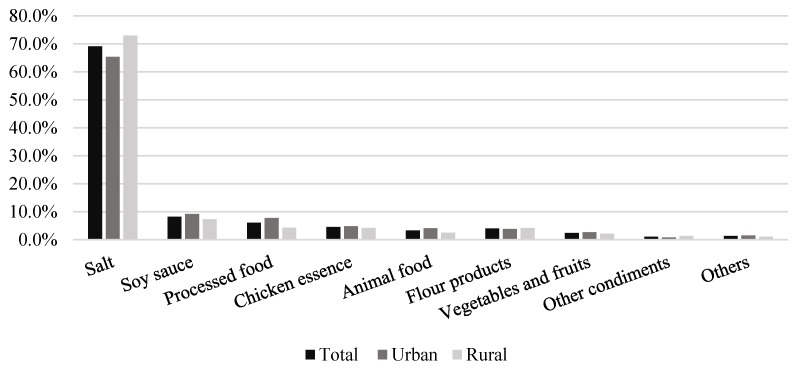
Proportion of sodium intake from various sources.

For the total sample, salt added by individuals when cooking/preparing a meal was the leading source of sodium, accounting for 69.2%. Soy sauce was the next highest contributor (8.2%), followed by processed food (6.0%) and chicken essence (4.5%). The proportion of sodium contributed by salt increased as age increased in both urban and rural subjects. On the contrary, the proportion of sodium from processed food decreased as age increased in both urban and rural subjects. Sodium contributed by processed food (7.7%) was a higher proportion of total sodium intake in the urban adults compared to the rural adults (4.3%), and the urban adults aged 18–49 years old obtained a higher proportion of sodium from processed food (8.1%) compared with the rural adults aged 18–49 years old (4.4%). Subjects from the north (7.1%) consumed a greater proportion of sodium from flour products compared with subjects in the south (1.4%) (Table 3, Figure 1).

## 4. Discussion

To our knowledge, this is the first study to describe, in detail, the dietary sodium intake, estimate the dietary sodium sources, and determine the prevalence of overconsumption among Chinese adults. Unlike previous studies [10,16,22], we observed sodium consumption among a wide range of adults. The sodium intake in rural subjects was higher than in urban subjects, and subjects from the north had a higher consumption level compared to subjects from the south. Otherwise, according to this study, the average intake of dietary sodium in Chinese was far in excess of the PI-NCD and the recommended level set by WHO, and salt was the main source of sodium among all recruited adults.

The sodium intake of recruited adults (5013 mg/day) was higher than the overall world average (4000 mg/day) and even exceeded the maximum recommended amount of sodium consumption by 90% and more [23]. Previous research, which consisted of a sample of Chinese adults (*n* = 6072) has already shown a concerning excessive sodium intake and an average consumption of sodium of 5.4 g/day [22]. Sodium maintains blood pressure, while consuming an excessive amount of sodium increases the risk of hypertension and NCD [4,24,25]. Although a recent study has shown that sodium consumption has been declining since 1982 in China [10], there are still some gaps comparing with the recommendations of the WHO [1]. Since salt added to food is the main source of dietary sodium, controlling salt intake is an important measure to reduce dietary sodium. In addition, further measures are needed to regularly limit and monitor salt/sodium intake. [22].

Sources of dietary sodium vary among countries. In Japan, the mean consumption of sodium is 4651 mg/day, and more than half of the sodium intake (63.2%) is obtained from soy sauce (34.6%), meats and eggs (20.4%), and vegetables and fruits (8.2%) [16]. In the United Kingdom, sodium intake is 3406 mg/day, and the main sources of sodium are bread and grains (20.0%), salted vegetables and fruits (9.8%), miso soup (9.7%), fish (9.5%), and salt (9.5%) [16]. The main sources of sodium in Australia are breads, cereal-based dishes, processed meat, and savory sauces [26]. The present study shows that the source of dietary sodium is different from other countries. The main source of dietary sodium in China is salt (69.2%) added to food during meal preparation. The proportion of dietary sodium contributed by salt is higher than in countries such as Japan, the United Kingdom, and Australia [16,26]. Soy sauce (8.2%), processed food (6.0%), and chicken essence (4.5%) are the next main contributors.

Salt is one of the main condiments in China, and the previous study shows that Chinese consumption of salt is the highest all over the world [27]. The present study shows that salt is the leading source of sodium, accounting for more than two-thirds (68.7%) of total sodium intake. Thus, controlling salt consumption is essential for reducing sodium intake, and the strategies of reducing salt intake are effective in many countries. In 1979, Finland took action to reduce salt intake by developing reduced-salt food products with the food industry and raising general awareness on salt and health [28]. As a result, the prevalence of hypertension and mortality of stroke decreased [28,29]. The salt intake dropped from 9.5 g to 8.6 g in four years since the British government took measures to improve food processed with salt [30]. In addition, countries such as Canada [31], Japan [32], and America [33] carried out salt reduction activities. In recent years, salt reduction activities have been carried out to reduce salt intake, such as limiting excessive salt added to food during cooking a home-cooked meal and reducing sodium intake when eating out (restaurant and canteen). At the same time, it is possible to reduce the added salt in food by adding natural seasonings to the food, which can improve its taste.

It should be noted that dietary sodium contributed by flour products ranked third for adults from the north, accounting for 7.1% of total sodium intake. Salt is an important component of flour products, since it increases flour products’ shelf-life and improves the taste [34]. Previous studies have shown that the amount of salt in bread from 397 mg/100 g bread in the United Kingdom to 1800 mg/100 g bread in Turkey [35]. Northern subjects consumed more sodium from flour products than southern subjects, which may be attributed to northern adults’ preference for noodles and bread. A longitudinal study of Chinese adults found that the consumption of flour products in the northern area was more than twice that in the southern area [36]. Thus, for northerners, public health education should be implemented to raise awareness of the effects of sodium in flour products, which will help to reduce the total dietary sodium intake.

With the rapid development of the economy, the transformation from a traditional dietary pattern to a modern dietary pattern accelerated, and the consumption of processed food represented by high sodium and high energy was on the rise [37,38,39,40]. The present study shows that dietary sodium contributed by processed food in the economically developed urban areas is 3.3%, higher than 1.6% in rural areas. This is consistent with the results of Mizehoun-Adissoda et al. [41]. The amount of dietary sodium contributed by processed food in rural areas is lower than that in urban areas, for the latter may be easier to access processed food than the former [42]. In recent years, urbanization has accelerated the Chinese market expansion of processed food [43], which has provided residents more opportunities to obtain processed food. Therefore, the total dietary sodium could be reduced by controlling sodium from processed food, and the department concerned should strengthen supervision over the labeling of sodium in processed food so that consumers can reasonably choose low-sodium food.

The young prefer modern food and processed food. The present study found that the sources of dietary sodium differ between age groups in both urban and rural subjects, and the amount of sodium contributed by processed food and animal food in the 18–49 years-old group was higher than in the elder age group. A study conducted in the US (*n* = 22852) also showed a gender difference in food sources of dietary sodium. Subjects aged 20–50 years old obtained a higher proportion of sodium from pizza, pasta, chicken, beef, and Mexican mixed dishes compared with subjects aged 50 years old and over [44]. In Spain, adolescents and adults consumed a higher proportion of dietary sodium from meat, processed meat products, and ready-to-eat meals than older people [45].

There were three limitations in this study. Firstly, we calculated the sodium provided by food according to the Chinese Food Composition Table. However, in the actual operation, since there was a lack of some kinds of processed food data in the Chinese Food Composition Table, and the composition of different brands of food may be different, we used similar foods to estimate the sodium content of such foods, which may be inaccurate for calculating dietary sodium intake. Secondly, the sodium contributed by eating out was calculated based on the food eaten at home, which could have led to an underestimation of total dietary sodium intake. A previous study showed that salt consumption provided by eating out is higher than that eating at home [46]. Thirdly, 24-h urinary sodium excretion is the gold standard for estimating sodium intake, but due to the large number of recruited adults in this study, the total dietary sodium was estimated from three consecutive days. The sodium results obtained in this study are higher than recalls combined with the household food weighing method instead of 24-h urinary sodium excretion; thus, the consumption of sodium may have been underestimated.

## 5. Conclusions

In conclusion, controlling the dietary sodium intake is a challenging and relevant public health goal as the status of Chinese sodium intake is more than twice as much as the recommended consumption set by WHO. Overconsumption of sodium is positively related to adverse health outcomes, such as hypertension, so efforts to reduce sodium from diets must be enhanced. The data of this study show that salt is the main contributor to total sodium intake; thus, conducting “action on salt China” as well as strengthening health education are key goals for public health authorities to reduce sodium intake.

## Figures and Tables

**Table 1:** Demographic characteristics of the 53,993 adults.

	Total Sample, *n* (%)	Urban, *n* (%)	Rural, *n* (%)
Age (years)			
18–49	25,309 (46.9)	11,393 (42.5)	13,916 (51.1)
50–64	18,320 (33.9)	9573 (35.7)	8747 (32.2)
≥65	10,364 (19.2)	5816 (21.7)	4548 (16.7)
Gender			
Male	24,777 (45.9)	12,053 (45.0)	12,724 (46.8)
Female	29,216 (54.1)	14,729 (55.0)	14,487 (53.2)
Geographic region *			
South	28,781 (53.3)	14,860 (55.5)	13,921 (51.2)
North	25,212 (46.7)	11,922 (44.5)	13,290 (48.8)
Highest level of education			
Primary school or less	22,226 (41.2)	7355 (27.5)	14,871 (54.7)
Middle school	27,145 (50.3)	15,257 (57.0)	11,888 (43.7)
College and above	4621 (8.6)	4170 (15.6)	451 (1.7)

*: Geographic region: northern and southern regions were divided by Qinling Mountain and Huaihe River.

**Table 2:** Usual dietary intake and the prevalence of excess intake of sodium (mg/day).

	Urban				Rural				Total			
	Mean (95% CI)	Median (p25, p75)	Over PI-NCD * (%)	Over WHO Standard (%)	Mean (95% CI)	Median (p25, p75)	Over PI-NCD (%)	Over WHO Standard (%)	Mean (95% CI)	Median (p25, p75)	Over PI-NCD (%)	Over WHO Standard (%)
	5056 (4887, 5232)	4563 (3418, 6153)	92.0	91.4	4966 (4705, 5228)	4435 (2977, 6296)	93.1	92.6	5013 (4858, 5168)	4505 (3219, 6211)	92.6	92.0
Age (years)												
18–49	4994 (4814, 5174)	4521 (3377, 6094)	91.2 ^a^	91.2 ^b^	4872 (4592, 5152)	4329 (2869, 6193)	92.6 ^a^	92.6 ^b^	4931 (4763, 5099)	4439 (3140, 6151)	91.9 ^a^	91.9 ^b^
50–64	5276 (5072, 5480)	4704 (3548, 6327)	94.0	92.9	5239 (4962, 5516)	4691 (3199, 6595)	95.1	94.1	5259 (5093, 5426)	4702 (3404, 6456)	94.5	93.4
≥65	4959 (4793, 5125)	4462 (3374, 5958)	92.3	89.1	5058 (4838, 5279)	4506 (3207, 6228)	92.2	89.8	5007 (4871, 5144)	4488 (3302, 6086)	92.2	89.5
Gender												
Male	5143 (4945, 5341)	4637 (3426, 6330)	93.7 ^a^	93.2 ^b^	5061 (4773, 5349)	4533 (2943, 6495)	94.5 ^a^	94.1 ^b^	5102 (4928, 5275)	4601 (3215, 6402)	94.1 ^a^	93.7 ^b^
Female	4975 (4821, 5129)	4480 (3410, 5957)	90.3	89.6	4868 (4626, 5110)	4343 (3008, 6045)	91.7	91.1	4922 (4780, 5063)	4422 (3224, 5994)	91.0	90.3
Geographic region												
South	4925 (4736, 5114)	4437 (3395, 5876)	92.2	91.6	4408 (4100, 4717)	3984 (2619, 5616)	93.0	92.5	4681 (4506, 4857)	4245 (3029, 5768)	92.5	92.0
North	5238 (4931, 5544)	4717 (3463, 6449)	91.9	91.2	5544 (5210, 5877)	4939 (3447, 6972)	93.2	92.8	5400 (5176, 5625)	4833 (3454, 6704)	92.6	92.1
Highest level of education												
Primary school or less	5223 (5022, 5424)	4687 (3526, 6327)	92.3 ^a^	90.9 ^b^	5063 (4796, 5330)	4482 (3058, 6389)	92.8	92.0	5118 (4933, 5303)	4562 (3240, 6371)	92.6 ^a^	91.6 ^b^
Middle school	5047 (4854, 5240)	4554 (3418, 6144)	92.5	92.0	4897 (4614, 5180)	4407 (2925, 6223)	93.5	93.3	4977 (4811, 5143)	4495 (3215, 6175)	93.0	92.6
College and above	4871 (4668, 5073)	4407 (3258, 5853)	89.8	89.7	4808 (4391, 5226)	4099 (2840, 6019)	91.2	91.0	4860 (4679, 5042)	4368 (3203, 5903)	90.1	89.9

*: Proposed Intakes for Preventing Non-communicable Chronic Diseases. ^ab^: Proportion of subgroups are significantly different (*p* ≤ 0.05).

**Table 3:** Proportion contribution of the main food groups to total dietary sodium (%).

			Salt	Soy Sauce	Processed Food	Chicken Essence	Animal Food *	Flour Products	Vegetables and Fruits	Other Condiments	Others **
Urban											
	Gender	Male	65.1	9.3	7.7	4.9	4.0	4.0	2.6	0.8	1.5
		Female	65.6	9.0	7.8	4.8	4.1	3.5	2.8	0.8	1.5
	Age (years)	18–49	64.8	9.3	8.1	4.7	4.2	3.9	2.7	0.8	1.5
		50–54	66.4	9.0	7.0	5.1	3.8	3.7	2.7	0.8	1.5
		≥65	66.6	8.7	6.9	5.2	4.0	3.6	2.9	0.8	1.3
	Geographic region	South	65.2	9.6	7.5	5.6	4.8	1.4	3.4	0.9	1.6
		North	65.5	8.6	8.0	3.8	3.2	7.0	1.8	0.6	1.4
	Highest level of education	Primary school or less	68.6	8.0	6.8	5.1	3.3	3.3	2.7	0.8	1.3
		Middle school	65.1	9.3	7.8	4.8	4.1	4.0	2.7	0.8	1.5
		College and above	65.6	9.0	8.7	4.5	5.3	3.9	2.7	0.6	1.7
Rural											
	Gender	Male	72.9	7.3	4.2	4.2	2.4	4.3	2.0	1.4	1.1
		Female	73.0	7.3	4.4	4.2	2.5	4.1	2.2	1.3	1.1
	Age (years)	18–49	72.4	7.4	4.4	4.4	2.5	4.4	2.1	1.4	1.1
		50–54	74.2	7.1	4.1	4.0	2.2	3.9	2.0	1.4	1.0
		≥65	74.7	7.1	4.1	3.6	2.4	3.9	2.1	1.2	1.0
	Geographic region	South	73.5	6.8	4.0	5.5	3.1	1.4	2.7	1.8	1.2
		North	72.4	7.8	4.7	2.9	1.7	7.1	1.4	0.9	1.0
	Highest level of education	Primary school or less	74.7	6.6	3.9	4.1	2.2	4.0	2.1	1.4	1.0
		Middle school	71.7	7.7	4.6	4.3	2.6	4.4	2.1	1.3	1.1
		College and above	69.6	8.7	5.4	4.3	3.3	3.7	2.2	1.7	1.2

* Animal food: including red meat and products, poultry and game, eggs, dairy, fish and shrimp. ** Others: including rice, potatoes, soybean and soybean products, and edible oil.
